# Cholera

**Published:** 1873-07-15

**Authors:** J. S. Jewell


### CHOLERA.

BEING THE SUBSTANCE OF A LECTURE DELIVERED TO THE SUMMER CLASS IN THE CHICAGO MEDICAL COLLEGE, BY J. S. JEWELL, M.D.

Your attention is called to this subject because of its importance, and because of the probability that the disease may prevail in various parts of the country this year, and because I entertain certain views in regard to its pathology and treatment which, if they are not new, are not common. In what I am to say I shall give you simply the *conclusions* at which I have arrived after a somewhat protracted study.

I. ITS CAUSE.—The true causes of disease are seldom positively known. We behold certain phenomena or *effects* which, taken together, constitute this or that disease. But the *causes* of the effects are seldom known—often unknown. However much this is to be regretted, it is no matter of surprise to those who are at all acquainted with the progress and philosophy of medicine. The above remarks are especially true for epidemic diseases, such as cholera is.

Much is said, I know, about bad air, bad food, bad water, etc., as *causes* of cholera. But these unhealthy agencies always exist the same, as a rule, when cholera does not prevail and when it does. Like causes produce like effects. If such agencies could cause cholera one year, they should every year; but they do not. It is true, no doubt, that such agencies may make it *worse* if it comes, or afford the reason why it prevails most in ill-ventilated localities, in large cities, where people are crowded together and have bad food, and live in dirty habitations, and are filthy in person. But they cannot be the essential and peculiar cause of the disease. Then what *is* the cause? It has, perhaps, never yet been positively discovered. But for various reasons I cannot give you to-day, we may *infer* something as to its nature and mode of action.

Judged from such a point of view, the cause of cholera is probably a vegetable microscopic organism, which appears occasionally—like the grape-blight, for example—and depends on conditions, atmospheric and otherwise, at present unknown.

This minute vegetable organism (or its spores) is supposed to be introduced into the blood in some way, in which it rapidly multiplies. It is further supposed, while circulating in the blood, to act on the nervous system, not unlike many powerful poisons of a vegetable origin are known to do.

You will remember that there are two parts to the nervous system, which differ from each other in some respects. One part, the cerebro-spinal system, is the immediate bodily instrument of the mind, the seat of true sensation, the spring of voluntary muscular motion, the organ of our mental acts. The other part, the sympathetic or vaso-motor system, is not the seat of true sensation, has but little if anything to do with voluntary muscular motion, and is not the organ of our mental acts. It is especially concerned in the circulation of the blood; in exciting and controlling the actions of the small muscular arteries—the heart in part—and in all those actions which are naturally removed from the influence of the will.

Though these things are so, these two parts (if we may so call them) of the nervous system are closely related, and within certain limits perform similar functions. Now, it is on this latter part of the nervous system the cause of cholera is supposed to act. To produce the effects we observe in cholera, and in view of what is now known in relation to the functions of the vaso-motor nervous system, it must act on it, as we may more fully see as I pass on.

As chloroform, for example, so acts on the cerebro-spinal system as to abolish true sensation, the power of voluntary muscular motion, and, as a rule, all mental action, and yet permits the circulation, respiration, etc., to go on much the same as in health, so the presumed poison of cholera, on the other hand. It acts on the vaso-motor nervous system, whatever else it may do, but spares the cerebrospinal nervous system, which is only secondarily affected. Consequently the mind remains clear, as a rule, sometimes even till death, amid the most signal wreck of what have been called the organic functions. The effect of the poison in question on the vaso-motor nervous system is greatly to diminish, or even destroy, its power and its sensibility, such as it has. Inasmuch as the action of the heart and small muscular arteries is as dependent on this nervous system as the voluntary muscles are on the spinal cord and brain, this loss of power and excitability in the vaso-motor nervous system is followed by weakness in action, or a sort of paralysis of the small muscular vessels, on the action of which the circulation of blood through the capillaries is almost absolutely dependent.

Immediately the action of the heart becomes feeble, and the capillary circulation in great measure fails. In consequence of this all of the functions dependent on a due circulation of the blood also fail in a measure. The skin becomes cold, because, unless a healthy amount of blood, well supplied with oxygen, is circulated, animal heat cannot be produced. The blood does not circulate in the small vessels of the lungs any better than those of the skin, and hence it does not absorb oxygen only imperfectly. Because the blood is not oxygenized it becomes dark, or what is called *venous.* This imparts a dark or livid hue to the skin, especially such parts as have most blood. All the functions comprehended under the title of nutrition suffer, or may even be abolished. On every hand, and for plain reasons, there is prostration of the powers of life, especially *organic* life.

Beside the foregoing phenomena, there are others yet to be noticed, most striking of all. They relate to the vomiting and purging. How can we explain these phenomena? Very easily, on the theory I have just stated.

The blood goes out from the heart to all parts of the body, through the arteries, until it comes to the smallest of all the vessels, the capillaries. In most parts of the body, after it has passed through the capillaries, it enters the veins and proceeds directly toward the heart. But in case of the stomach and bowels this is not so. The blood is sent to the stomach and bowels, especially the lining mucous membrane of the same, by the arteries, as to other parts. It enters and passes through the capillaries into the veins as elsewhere. But here the similarity to other parts ends. The blood, once in the veins, does not go to the heart direct, as in most other parts. It is collected into what is called the *portal vein*, which, instead of going toward the *heart*, goes toward the *liver*, and there divides again in the liver up into thousands of small vessels, which terminate in the capillaries of the liver. It thus happens that the portal vein has capillaries at both ends. Thus the blood from the alimentary canal, which has had to struggle as it were through *one* set of capillaries in the mucous membrane of the bowels, must now pass through *another* set, in a massive, solid organ like the liver is. If the heart and small muscular arteries have in great measure lost their power to circulate the blood through *one* set of capillaries, much more have they lost it to propel the blood through *two* sets of capillaries.

The blood from the arteries, at each beat of the heart, goes to the intestinal canal, but can get only imperfectly beyond the portal vein. This vein, and the capillaries back of it, and soon the arteries of the mucous membrane of the alimentary canal, become greatly distended and stretched with blood. Every heart-beat renders this state of things worse. In a short time, if this state shall continue, two things must happen:

1. There will be a loss of balance in the circulation. Too much blood will be collected in the vessels of the stomach and bowels, and by just so much will there be too little elsewhere, especially in parts remote from the heart, as the skin and exetremities. They become pale and shrunken, because they have so little blood. It is penned up, as it were, in the vessels of the stomach and bowels, and each pulsation of the heart makes things worse until recovery begins.

2. The blood-vessels—especially the capillaries and veins of the mucous membrane of the bowels—become exceedingly full of blood—are distended and swollen. At last, from the expansive pressure within, and the thin-tense state of the walls of the vessels, the watery part of the blood—like sweat from the skin on a hot day—begins to exude from the surface of the mucous membrane lining the bowels. It soon, in bad cases, fills the bowels with the serum of the blood. This is soon vomited up or purged away, constituting what is called the "rice-water" discharge. In this way the vessels are partially relieved from their over-distension. But still the heart sends blood to the seat of the disorder, and in this way the distension, which is relieved by exudation, is maintained, and the unhealthy process goes on. Nearly eight-tenths of the mass of the blood, and more than that of its volume, is water. With the rapid discharge from the alimentary mucous membrane of the watery part of the blood, and no available means to supply it back again, the blood becomes viscid or thick. This makes the circulation of the blood still more difficult, especially in massive organs like the brain, spinal-cord, kidneys, liver, etc. The body becomes, in bad cases, greatly shrunken or emaciated, and suddenly so, from the loss of so much fluid. The patient has inexpressible thirst, and drinks freely, if permitted, only to vomit it up again. And a moment's consideration will show that the stomach and bowels are in such a state as to render the absorption of fluids, and indeed anything else, all but impossible—because the vessels of the mucous membrane of the bowels are already distended to such a degree that the contained fluid is oozing out in little streams through the sides of the vessels. This is why drinks or medicines of any kind do so little good in the stage of collapse or depression in cholera. They are not absorbed, and, in the nature of the case, cannot be. If the case is a bad one, these things go on rapidly from bad to worse, and the patient—in a cold, pulseless, shrunken state—dies quietly, as a rule. But if not so bad, especially if appropriate treatment is applied, the patient begins soon to improve.

Such, in brief, are my views as to the pathology of cholera. They have not been hastily formed or adopted. They are not the views commonly held. Their value will depend on the practical use that can be made of them. A rational pathology should indicate plainly a correct line of treatment.

Let us see what the practical deductions are that may be drawn from the foregoing views of the pathology of the disease. If the cause be such as above supposed, all we can do at present practically to prevent the disease is to avoid anything or everything which would tend to impair the power and health of the body. Eat plain, fresh, digestible food; take simple drinks; avoid bad air, whether in or out of doors; do not become exhausted by too much exercise, either physical or mental; get plenty of sleep; do not be afraid of the disease, and in this way enfeeble the powers of resistance to morbid influences. By such means one may not escape the cause of the disease, but he may acquire and preserve sufficient power to resist its action successfully; and these things are found to be true in great measure. So much for the *prevention* of the disease.

I will come now to *curative* measures. I will confine my remarks to the treatment of the stage of depression or collapse, because it is incomparably the most important. If a patient passes this period he is justly considered as comparatively safe. Now, what treatment do our views of pathology dictate?

1. That medicines given by the mouth cannot be absorbed, in most cases, only imperfectly; and by just so much are useless. If a fluid could be absorbed from the mucous membrane of the stomach, why not absorb that which is already in the stomach, but which is vomited so freely up? There may be cases in which these remarks do not hold good, but they must be rare. If these observations are just, we must look in some other direction with our medicines. We need prompt and decisive action. We need to have something introduced into the blood to act, if possible, both on the blood and as a powerful stimulant to the vaso-motor nervous system. Now, how shall we get it there, and what shall it be?

2. What can we use that will act powerfully and certainly on the vaso-motor nervous system, and also on the blood, or the assumed cause in it? As regards the first point, I would name *strychnia* and *caffeine*, and also *morphia*. But the first two agents stand first in rank, in my judgment. They are known to be powerful and reliable nerve stimulants. They should be used by means of the hypodermic syringe. The sixteenth to the fortieth of a grain of strychnia may be given in combination ; one to three grains of caffeine, or the eighth of a grain of morphia ; or they may all be given at the same time, and in the same solution, with advantage. These hypodermic doses should be given at least once an hour, with intermediate doses of the caffeine. The alkaloids may be rubbed in distilled or rose-water, adding, generally, three to five drops of acetic acid to the ounce of water, to insure or aid in making the solution. Placed beneath the skin, in the areolar tissue, if the circulation has not absolutely stopped, there is every reason to expect the medicine will be absorbed. In this way you may certainly know how much has been taken; and it cannot be vomited up. This point in treatment—viz. : the sub-cutaneous employment of the remedies named—is one of capital importance, and deserves a most persevering and thorough trial, whatever else may be done. I can hardly speak with sufficient emphasis here. I fully believe, and not without pretty good reasons, that the stage of collapse may be more successfully met in the way indicated than in any other known. Let this course then be faithfully tried—all the more so when we see how patients are carried away on every hand, even under the best modes of treatment ordinarily pursued.

Beside the agents already recommended, I would use one other ; and I am guided to its use solely by my views of the pathology of the disease. If the cause of the disease be as has been supposed, then it would seem some of the antizymotics could be employed with advantage. In this relation I would recommend the use, hypodermically, of carbolic acid, in a solution of suitable strength. It has been already used with very decided success in miasmatic or intermittent fevers, and in the same way. I would expect it to destroy or check the production of the supposed microscopic organisms in the blood.

It might be used, also, as a sort of prophylactic, even by the mouth, in connection with suitable hygienic regulations. Beside these internal means, I would use most faithfully the external application of heat—in no small or fitful way, but severe frictions—continued with only brief intervals, if any at all, until the patient exhibits unmistakable signs of recovery or death. I would use stimulants externally freely, especially the alcoholic. But, to be of service, these external means must be employed unfalteringly and heroically, in bad cases. In this way the circulation can be greatly facilitated in the surface and extremities, and no small portion of the blood won from the abdomen or alimentary mucous membrane, and in this way the volume of the blood in a measure retained until the vaso-motor nervous system has been in some measure restored by stimulation, and, in consequence, the circulation which depends on it.

Such, in brief, are my views in relation to the pathology and treatment of cholera.

---

Treatment of the Inflamed Breasts of Nurses.—The method here recommended is so simple that no one need hesitate to adopt it, provided he is called in before the mischief has reached a certain degree of development. It is wellknown that engorgements of the mammary glands are frequently caused by chapped nipple. The inflammation of the skin extends directly into the ducts, exudations take place by which some of the ducts are plugged up, the milk is pent in, and hence the engorgement. If, now, in such a case, the breast be surrounded with the hands, and pressure made in the direction of the nipple, a thin, transparent, whitish vesicle is caused, by the milk accumulating behind the closed orifices of the ducts. It is necessary, then, to do this, and, having done it, the next thing is to prick the vesicle with a needle, to remove any epithelial scales which may be present, and to apply the infant. If time has not been lost unnecessarily, the relief is almost immediate, and pain and tumefaction disappear in a few minutes; but even when it is otherwise, the relief is very marked, and by repeating the process a few times, the sufferer is relieved altogether.—*Southern Medical Record*.

